# Barriers to the Integration of Care in Inter-Organisational Settings: A Literature Review

**DOI:** 10.5334/ijic.3068

**Published:** 2018-01-16

**Authors:** Carolin Auschra

**Affiliations:** 1 Freie Universität Berlin, Department of Management, Boltzmannstr. 20, 14195 Berlin, DE

**Keywords:** inter-organisational collaboration, networks, barriers, integrated care

## Abstract

**Introduction:**

In recent years, inter-organisational collaboration between healthcare organisations has become of increasingly vital importance in order to improve the integration of health service delivery. However, different barriers reported in academic literature seem to hinder the formation and development of such collaboration.

**Theory and methods:**

This systematic literature review of forty studies summarises and categorises the barriers to integrated care in inter-organisational settings as reported in previous studies. It analyses how these barriers operate.

**Results:**

Within these studies, twenty types of barriers have been identified and then categorised in six groups (barriers related to administration and regulation, barriers related to funding, barriers related to the inter-organisational domain, barriers related to the organisational domain, barriers related to service delivery, and barriers related to clinical practices). Not all of these barriers emerge passively, some are set up intentionally. They are not only context-specific, but are also often related and influence each other.

**Discussion and conclusion:**

The compilation of these results allows for a better understanding of the characteristics and reasons for the occurrence of barriers that impede collaboration aiming for the integration of care, not only for researchers but also for practitioners. It can help to explain and counteract the slow progress and limited efficiency and effectiveness of some of the inter-organisational collaboration in healthcare settings.

## Introduction

Leading institutions, practitioners and researchers have reached a consensus that health service delivery profits from integration [1,2,3,4] “across time, place and discipline” [5, p. 1]. Integrated care in its various forms can produce benefits such as quality enhancement, increased system efficiency and cost reduction, higher client satisfaction, and better access to care [1,6]. It is of importance to consider that the integration of care can be achieved by employing different forms of governance [3], ranging from the integration of tasks within organisational hierarchies (e.g. inter-professional collaboration within a single organisation such as a hospital) through collaborative inter-organisational relations [e.g. in service provider networks, see 7] to more market-oriented forms of coordination (where integration can be reached on a short-term, contractual basis). Thus, integration can help to coordinate previously separated tasks of care provision not only across professional or sectoral, but also organisational boundaries [3,8].

More often than not, the integration of care faces barriers [8,9,10,11] caused by contextual, institutional and professional factors in different domains of integrated care [1]. This paper, based on a systematic review of the literature, puts an emphasis on barriers to the integration of care in inter-organisational settings as one of the governance forms (market vs. inter-organisational collaboration vs. hierarchy). Inter-organisational collaboration is important in this regard, as many patients require a mix of services delivered by multiple, often formally and legally independent providers [1,12]. Beneficial practices of inter-organisational collaboration that help to integrate care include, for instance, the mutual exchange and transfer of information and knowledge, enhanced trust between providers, and the creation of synergy effects [13,14]. Inter-organisational collaboration can thus reduce fragmentation within healthcare systems and provide the potential to generate innovation in healthcare delivery (e.g. by bringing together complementary competences). Examples in different countries show the importance of inter-organisational collaboration for the delivery of integrated care. For instance, in the U.S., community-based health and human services are often delivered by networks of independent providers [15]. Additionally, accountable care organisations which can be found in the U.S., but also in countries like Germany, involve inter-organisational collaboration [16,17]. The British NHS has experimented with the integration of care through inter-organisational collaboration since the 1990s [8], as have the Nordic countries [18] and the Netherlands [8]. Understanding barriers that impede the development of collaborative inter-organisational relationships can promote the successful implementation of integrated care in such settings.

Despite their various potential benefits, many inter-organisational collaborations fail [according to 19 around 50–70%], and the implementation of collaboration proves to be a managerial challenge. Some scholars argue that a lack of common goals or leadership inhibits collaboration [20]. Others point to regulative constraints that many actors have already experienced while experimenting with collaboration [21]. Although such barriers are mentioned in various studies of integrated care in inter-organisational settings – partly as the main focus and partly as a by-product while elaborating on other facets of inter-organisational collaboration – so far no systematic review of the relevant literature has been compiled. Various literature reviews focus on inter-organisational collaboration and networks in different industries [22,23,24], but few of these explicitly addresses barriers [12,25,26], often with a very indication-specific focus on healthcare settings. Several empirical works address barriers to the delivery of integrated care in inter-organisational settings, but mostly focus on aspects specific to their case [11,27] and lack theoretical embedding.

Further attention is called for to barriers to the integration of care in inter-organisational settings, as there is a significant gap between what “could” be possible in collaborative practice and what actually is achieved within most inter-organisational relationships. Such a focus is especially important, as some barriers occurring during inter-organisational collaboration are particular to this governance form due to the existence of the inter-organisational domain, where, for instance, formally autonomous and culturally different organisations collaborate. The aim of this paper is therefore to develop deeper insights on barriers that impede integrated care delivery in inter-organisational settings by reviewing previous research on barriers to inter-organisational collaboration in healthcare. What is more, this study also aims to generate insight into how such barriers operate.

Thereby, the study offers the following contributions: first, it deepens our understanding on barriers to the integration of care in inter-organisational settings by providing a systematic overview on several kinds of barriers that can occur, on their contextual embedding, as well as underlying mechanisms that lead to their existence. This helps to explain why some inter-organisational collaborations that aim for the integration of care make slow or no progress. Second, this systematic literature review can guide further empirical research on the occurrence of barriers and their causes. Furthermore, it can help practitioners engaged in the planning or implementation of inter-organisational, integrative health care services to avoid or overcome such barriers by promoting awareness and enabling more reflective action.

## Theory

According to Kodner and Spreeuwenberg [1], integration in healthcare “is a coherent set of methods and models on the funding, administrative, organisational, service delivery and clinical domains designed to create connectivity, alignment and collaboration within and between the cure and care sectors” (p. 3). This definition gives a reference point to two important concepts to which this paper refers: inter-organisational collaboration and domains relevant for the practice and the scientific analysis of integrated care.

### Inter-organisational collaboration in health service delivery

The present review focuses on inter-organisational collaboration as one governance form (beside markets and hierarchies) that enables the integration of care [3]. Inter-organisational collaborations in healthcare appear in several forms, e.g. as dyadic relationships between two partner organisations or as inter-organisational networks, implying relationships between at least three partners [24]. The variety of such collaborations in healthcare includes, among others, healthcare alliances [28], urban healthcare-delivery networks [15], digital health platforms that rely on interorganisational collaboration [29], and regional networks of service providers that negotiate population-based care contracts [16]. They can take on – following the integration needs of care – several directions: vertical collaboration along the chain of health service delivery, including, for instance, collaboration between providers of primary and secondary care, or horizontal collaboration between organisations of the same kind, e.g. between general practitioners [6,8,14]. Inter-organisational collaboration differs from intra-organisational collaboration that takes place *within* one organisation, e.g. between different professions, team members or across teams [30]. Obviously, inter-organisational collaboration can include or overlap with inter-professional and intra-organisational collaboration, especially in the context of integrated care.

Given the various forms of inter-organisational collaboration, a definition needs to cover their specific, common characteristics and also span their differences. A very broad definition in organisation theory describes inter-organisational collaboration in the middle of a continuum delimited by market and hierarchy as “a cooperative, inter-organisational relationship that is negotiated in an ongoing communicative process, and which relies on neither market nor hierarchical mechanisms of control” [31, p. 323]. Inter-organisational collaborations – as opposed to market or hierarchical relationships – feature certain characteristics: firstly, partners within the inter-organisational relationship follow either a common goal or purpose [20,23]. Secondly, the organisations involved stay formally independent and autonomous [32], but are obligated to their partners. Thirdly, the relationships allow for and result from exchange [33], e.g. of information, resources, activities and capabilities, and include social interaction [24]. Fourthly, the partners follow certain rules, norms, and structures within the relationship – rendering the relationship either formal or informal [34]. Fifthly, the participants of inter-organisational relationships can also be competitors, being for-profit, and/or public, non-profit organisations [23]. In consequence, not just any inter-organisational relationship between two or more organisations [32] can be considered an inter-organisational collaboration; it all depends on the “collaborative quality”, which, admittedly, is not easy to create and maintain [14]. The perception of the main elements of this collaborative quality varies within the literature reviewed, but there is agreement that inter-organisational collaborations differ significantly from market and hierarchical relationships regarding their content and governance of interaction.

It is important to note that, in contrast to the rather static definition of inter-organisational collaboration as a governance form between market and hierarchy, each inter-organisational collaboration underlies a dynamic, context-dependent, and history-laden process. Taking this view into account, inter-organisational relationships undergo an evolution, ranging from their initiation and formation to their development and then to possible dissolution [22,23,35,36]. Furthermore, when analysing inter-organisational collaborations, it seems necessary – due to their nature– to be aware both of the structures (e.g. of the collaboration itself, within its context) and of the actions of the collaborating partners [37].

### Domains of integrated care

Kodner and Spreeuwenberg (2002) have proposed five domains, representing certain fields of social action that are relevant for the integration of care, thereby also applying to the integration of care in inter-organisational settings. They describe these domains as ranging from the macro to the micro level of analysis: the domains of administration, funding, organisation, service delivery and clinical practice [1]. They can provide a helpful framework for the analysis of barriers to the integration of care in inter-organisational settings and are described in the following in greater detail.

One can argue that the most extensive domain affecting the integration of care is *administration*, also including regulations, on a very macro or environmental level of analysis. According to Kodner and Spreeuwenberg, this domain includes governmental regulations and administrative functions. Additionally, such regulations can also include historically grown institutions not set up by the government, but established by habit and/or through other actors [38]. As a second domain, *funding*, which is often dependent on the aforementioned administrative domain, heavily affects the integration of care [1], e.g. by either providing reimbursement for coordination practices or not. A third domain relevant for the integration of care is the *organisational domain*. Within this domain, characteristics of and practices within single organisations can play an important role, e.g. in intra-organisational teamwork. A fourth domain, *service delivery*, includes and is affected by factors such as staff training, inter-personal relationships between professionals and the distribution of responsibilities and tasks. Therefore, this domain touches a more micro, individual level of analysis. The fifth domain relevant for the integration of care is the *clinical domain*, involving, for instance, common professional languages, agreed understandings, practices and standards related to certain diseases, and ongoing communication with patients [1]. As the focus of this review is on collaboration across organisational boundaries, it seems feasible to add a sixth *inter-organisational domain* that accounts for the peculiarities of inter-organisational collaboration, e.g. its specific governance mechanisms such as, e.g. reciprocity between autonomous organisations. This is also the domain where the management of a collaboration can influence its outcomes [20]. Thus, the analysis of barriers to inter-organisational collaboration may benefit from a clear distinction between the organisational and inter-organisational domain.

### A conceptualisation of barriers

Barriers are not only an issue regarding the subject of inter-organisational collaboration, but also regarding innovation, (strategy) implementation, and organisational change. Surprisingly, although often used, the term “barrier” is seldom defined. Barriers represent obstacles or difficulties of a material or an immaterial nature that individuals or organisational actors need to overcome in order to achieve their aims [for definitions of barriers see e.g. 39,40]. Despite linguistic differences, the terms barrier, impediment, hurdle or obstacle are often used interchangeably [39]. It is important to note that barriers do indeed represent obstacles, but obstacles which can be overcome, often in a gradual, processual way. A barrier itself can show up as a symptom of one or more underlying causes that constitute the barrier [41]. Some authors argue that the terms “barrier” and “facilitator” describe two sides of the same coin; e.g. that the absence of a barrier or its opposite (e.g. good vs. poor management) can facilitate an implementation process [10,12]. However, such a view contains pitfalls, as the mere elimination of the factor that causes a barrier does not guarantee that a practice hindered by this barrier will take place. Often, additional facilitators are necessary to enable, for instance, the successful integration of care.

## Methods

**Figure 1:**
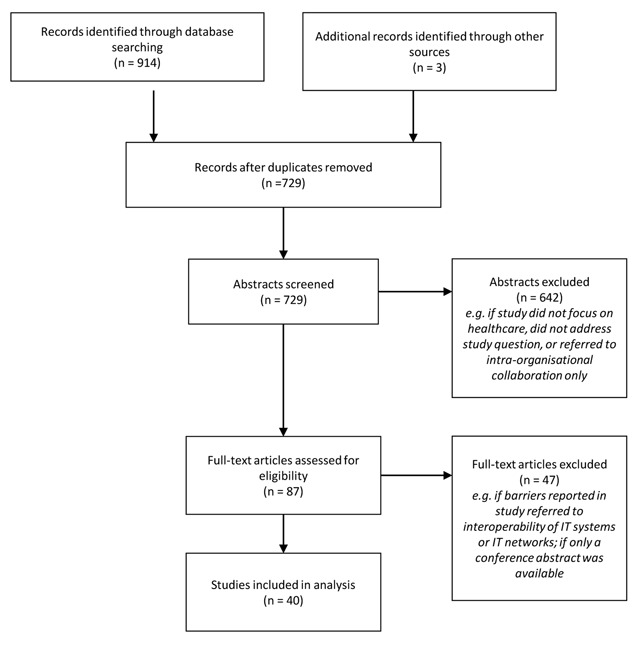
Flow chart of the identification and selection process.

To identify empirical and conceptual work that elaborates on barriers to inter-organisational collaboration in healthcare, a systematic review of literature was undertaken. The methodological approach used in this paper is informed by previous similar studies in the field of research on inter-organisational relationships and networks [24], integrated care [42,43], and – to improve transparency and replicability – is based on the PRISMA-guidelines for reporting on systematic reviews [44]. Figure 1 gives an overview of the review approach.

### Literature search

The first step was a systematic literature search. This review focuses on academic peer-reviewed articles in English-language journals that were retrieved from the databases PubMed (https://www.ncbi.nlm.nih.gov/pubmed); Cochrane Library (http://www.cochranelibrary.com/); Web of Science (https://apps.webofknowledge.com); and via the resource hoster EBSCOhost (https://www.ebscohost.com/) Business Source Premier, Communication Source, EconLit, ERIC, MEDLINE, PsycARTICLES, PsycCRITICS, SocINDEX, and Academic Search Ultimate. The date of publication was unrestricted (up to August 2017) and the search covered all disciplines available in the database (e.g. healthcare, management and organisation theory, economics and sociology). This approach enables a systematic review, although the analysis underlies some restrictions: monographs or chapters in edited volumes were intentionally omitted, as they are not listed systematically in data bases and may show quality constraints due to a lack of peer-review. Nevertheless, this analysis offers insights into the most important aspects of the academic discourse on barriers that impede the integration of care in inter-organisational settings.

Different search terms were applied, obtained from the definitions of inter-organisational collaboration, barriers, and integrated care, and including various synonyms. They were run for matches with synonyms for “inter-organisational” to exclude, for instance, research on neuronal networks (for an overview of the search terms and their applications, see Table 1 in the appendix). These initial keywords were chosen to cover as many relevant articles as possible. At the same time, by limiting the application of some of the search terms to titles and abstracts, the screening of ten-thousands of mostly irrelevant articles was avoided. In a final step of the search strategy, a PubMed-search was conducted with MeSH terms covering the integration of care (intersectoral collaboration; cooperative behaviour; public-private sector partnerships; community networks; delivery of health care, integrated) which replaced other synonyms for integrated care. It revealed six additional hits. This first search produced a total of 914 potentially relevant hits. To increase the consistency and robustness of the findings, monographs [20] and edited volumes [32], with a similar focus and which were mentioned in the articles, were also surveyed. Three additional records were identified through other sources, e.g. talks with experts. Duplicates in the identified records were removed; 729 articles then remained.

### Inclusion and exclusion criteria

In a second step, irrelevant hits were sorted out from the potentially relevant articles by reading the abstract of each article. Articles not relevant for the study were excluded. Studies were screened for their fit with the theoretical conceptualisation of inter-organisational collaboration and integrated care as presented in the theory section. Empirical studies that did not focus on integrated care settings were excluded, as they mainly elaborated on collaborations with goals different to that of health service delivery. Further reasons to exclude studies were if a study solely reported on barriers within single organisations (e.g. on inter-professional collaboration, but not with a focus across organisational boundaries), and articles not addressing the study question in other ways (e.g. studies referring to vector-borne disease outbreaks or barriers in IT-networks). This narrowed the scope of articles to 87 potentially relevant ones.

In a third step, all the remaining articles were read in depth in order to determine whether they were suited to explain the occurrence of barriers that impede the integration of care in inter-organisational settings. To stay in line with this focus, further studies were excluded, e.g. when they referred only to barriers to the interoperability of IT systems or IT-networks. Abstracts of conference presentations with unavailable full texts were also excluded.

### Analysis

The use of this procedure led to the identification of 40 relevant articles that matched the predefined search criteria and constitute the core of this review (see Table 2 in the appendix). They were classified according to different criteria which had been generated deductively [24] and inductively (e.g. by defining the focus of this review and reviewing the body of knowledge on this topic). Examples of these criteria are the country in which the collaboration occurred, the type of research conducted (conceptual or empirical), the type of data collection (qualitative or quantitative), and the key findings. During further analysis, the author reflected that not all criteria were central to the following argumentation (e.g. theoretical approach of the articles, barriers cited within these articles). That is why only a subset is presented in the table. These criteria help to get an overview of the applied research methods, and theoretical and contextual embedding of the reviewed studies. Afterwards, the barriers identified in the selected articles were coded by the author to identify the different types of barriers reported, following the approach of thematic synthesis that was already applied while doing reviews focusing on barriers [45]. The articles were first coded line by line with rather descriptive codes, representing barriers that occurred. A repetition of the coding after three months increased the reliability of coding. The emerging types of barriers were then assigned to six categories representing analytical themes, derived from the domains regarded important for the integration of care as proposed by Kodner and Spreeuwenberg [1]. During coding, the author was sensitive to potential sources of barriers, which could be rooted both in structure and in agency [37]. Additionally, potential relations between barriers reported within singular studies were visualized [46].

## Results

The issue of barriers that impede inter-organisational collaboration in health service delivery has been addressed in the selected sample of articles in both an empirical (35 studies) and a conceptual way (5 studies, three of them literature reviews, however with the main focus not being on barriers to inter-organisational collaboration). The research design of the empirical studies was mostly qualitative; only two used a quantitative approach and one a mixed-method design. Some studies addressing barriers to inter-organisational collaboration use no theoretical conceptualization at all [e.g. 47], and no general approach exists to conceptualize barriers that impede inter-organisational collaboration. Some studies rely on leadership concepts [48], neo-institutional theory [49], a complex adaptive systems perspective [50], professional identities [51], or innovation approaches [27]. This leaves room for further conceptual work, as the last section will show.

**Figure 2:**
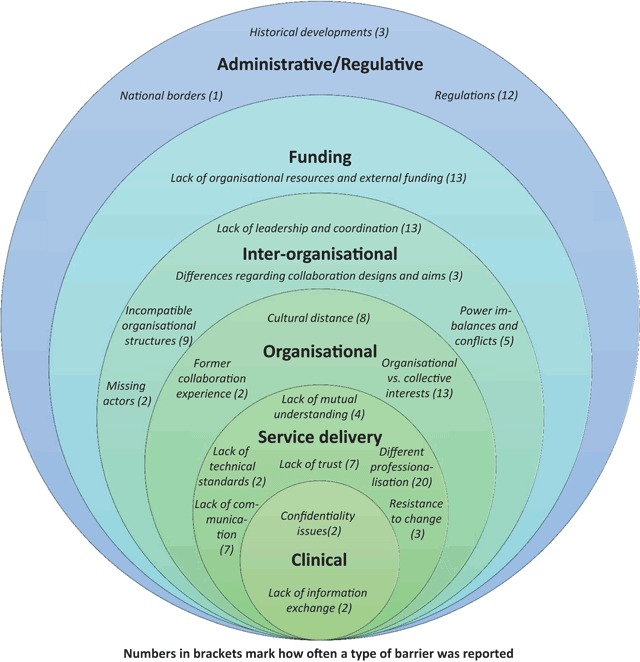
Barriers to the integration of care in inter-organisational settings.

Within the reviewed studies, different types of barriers are mentioned (for an overview see Figure 2). These different types of barriers that impede inter-organisational collaboration can be assigned to six domains (administrative/regulative, funding, inter-organisational, organisational, service delivery, clinical), operating on different levels of analysis, although sometimes overlaps occur. The consideration of different levels is useful as conflicts on the level of service delivery, for example, can also influence collaboration on the inter-organisational level [52], and vice versa.

Figure 2 also shows how often a certain type of barrier was reported in the reviewed studies (numbers in brackets). The barrier “different professionalisation” was reported most frequently (n = 20), followed by “lack of leadership and coordination” (n = 13) and “organisational vs. collective interests” (n = 13). In sum, most of the barriers mentioned (regarding the amount of the types of barriers and sum of the reported numbers in each domain) are assigned to the domain of service delivery, followed by the inter-organisational domain. This could be an indicator that many reasons for the slow progress or even failure of the delivery of integrated care across organisational boundaries can be found in the last domain. However, this interpretation may well underlie a bias, as we do not know if the reviewed studies illustrate all existing barriers that hampered a collaboration or if researchers maybe also intentionally (e.g. due to the research question) or unintentionally (e.g. due to observation bias) focused on certain barriers. Also, the range of the cases included (omitting for the most part, for instance, inter-organisational collaborations in the third world) involves limitations to this interpretation. The following paragraphs give an overview of the barriers analysed.

### Barriers related to administration and regulation

*National borders:* Borders of neighbouring territories can work as barriers to the integration of care in inter-organisational settings, especially by causing administrative or regulatory differences due to different healthcare systems and languages [49]. When inter-organisational collaboration stretches over a wider geographical distance, “differences in the meaning and use of relevant concepts between countries and regions” [53, p. 950] may also occur.

*Historical developments:* Historical developments and critical junctures, often on the macro level, influence the behaviour of organisational and individual actors. Regarding inter-organisational collaborations, some regions seem to use cooperation practices more than others (e.g. when comparing the integration of care within different countries). Collaboration between organisations belonging to regions with a long history of the fragmentation of care, e.g. in regard to specialization and ideology, may be difficult [54]. Furthermore, in some cases, the surrounding context traditionally sets no incentive to inter-organisational collaboration – e.g. by failing to offer financial support for collaboration between hospitals and other health service providers [18,50].

*Regulations:* Existing regulations can impede inter-organisational collaboration, either by forbidding it or making the implementation process extremely complicated, costly and time-consuming for the partners involved [12]. For instance, the legally required focus on bureaucratic procedures by organisations within the public sector can slow down collaboration [10]. Legal requirements can also hamper the information exchange (e.g. of patient data) needed to enable planned inter-organisational collaboration, or they can provide obstacles to the pooling of budgets in the public sector [8,55].

### Barriers related to funding

*Lack of organisational resources and external funding:* Sometimes organisations lack the resources needed to initiate and develop inter-organisational collaboration [e.g. 47]. Organisational managers are often unable to invest necessary resources like time [50] to develop a collaboration, as they are needed elsewhere. Furthermore, they fear cost shifting connected to the entry into an inter-organisational collaboration, e.g. through decreased governmental support in the public sector [56]. A general lack of funding for collaboration, i.e. in the public sector, is perceived as a main barrier to inter-organisational collaboration and causes high uncertainty for actors willing to collaborate [9].

### Barriers related to the inter-organisational domain

*Lack of leadership and coordination:* Proper leadership is important for conducting collaborative activities [10,20]. Inter-organisational collaborations often involve various stakeholders with different aims. If coordination between them is not conducted properly – for instance if organisational leaders start to protect their territory against the collaboration [12] the progress of collaboration can be affected considerably. That can be the case also when more planning takes place than implementation [56], which can lead to “overprocessing” [57] without any output on the level of care. A lack of leadership often causes uncertainties [11] and thereby hampers the further development of the inter-organisational collaboration.

*Missing actors:* The lack of important actors can be a barrier to successful collaboration in a certain nexus of health service delivery [12]. For instance, the failure to include a local hospital in a network of integrated care can cause difficulties. Especially before the formation of an inter-organisational collaboration, one facet of this barrier is the lack of knowledge about potential partner organisations. Due to the complexity of health service delivery and possibly insufficient mutual awareness of potential partners, their abilities and existing relationships can be limited [50]. In their study Tsasis and colleagues report on a healthcare professional who states that he does not necessarily understand exactly how other organisations in the same community contribute to the care of clients.

*Power imbalances and conflicts:* Perceived or real power asymmetries can become a strong barrier to inter-organisational collaboration and affect collaboration outcomes [23,58]. For instance, the dependence of one organisation on another can work as a barrier if the more powerful organisation does not provide the necessary input [59]. In case of power imbalances organisations often start to defend their own resources and authority, which often leads to power conflicts [57,60]. Power imbalances can also slow down planning and committee work in health service networks [58].

*Differences regarding collaboration design and aims:* Differing expectations about the gestalt and vision of an inter-organisational collaboration can also lead to controversies during network development [12,47], hampering further progress. For instance, Dinesen and colleagues [61] show that hospital and district nurses are sometimes unable to develop a common network vision, as they lack knowledge of each others’ competences.

*Incompatible organisational structures:* Differing organisational structures and processes can impede inter-organisational collaboration, e.g. by hindering common meetings due to different working arrangements [27]. Furthermore, within different organisations often divergent formal timetables and time horizons [47], different decision-making structures [56], and different views about employment, accountability and hierarchies [55] exist, affecting inter-organisational collaboration.

### Barriers related to the organisational domain

*Organisational vs. collective interests:* Within inter-organisational collaboration, different organisations with divergent goals and interests work together. Organisational goals do not need to overlap with collective goals [28]. Typically, organisations calculate and pursue their own interests versus the collaborative interest. If these interests are conflicting, barriers impeding the inter-organisational collaboration emerge and conflicting agendas arise. For instance, if a (mandated) collaboration threatens the political and economic interests of an organisation involved, it can be very reluctant to collaborate [62]. Differing organisational interests become more controversial if participants of inter-organisational collaboration are part of a “quasi-market”-relationship including the enactment of competition [56]. Taking part in an inter-organisational collaboration also implies a loss of organisational autonomy, e.g. if shared resources and joint planning require common decision-making processes, that can become problematic if organisational and collective interests do not overlap or even conflict [20,63]. When organisations start to protect their interests very strongly within an inter-organisational collaboration, this can lead to the situation that nobody will take on responsibility for common issues [18].

*Cultural distance between organisations:* Organisations develop their own specific cultures, which can create barriers to inter-organisational collaborations if organisations are not capable of managing these differences. Different orientations and norms result from various underlying cultural and institutional logics (e.g. regarding the meaning of time and ways of working). An individualistic working culture in one partner organisation is an example of how cultural distance can impede inter-organisational collaboration [18]. Cultural differences between organisations can affect various areas that are relevant for inter-organisational collaboration, e.g. the processes of decision making or the handling of clients [20,63].

*Former collaboration experiences:* Former cooperation experiences – either with a present partner or with others – influence both the willingness of organisations to collaborate and also their behaviour within existing collaborations. If they have gathered experiences from former collaborations, organisations assess cooperation outcomes differently [47]. Bad experiences in a former or an ongoing cooperation can be a drawback, leading, for instance, to behavioural reservations towards future collaboration among the employees.

### Barriers related to service delivery

*Lack of technological standards:* Especially inter-organisational collaboration that requires the use of common IT-infrastructure faces formidable challenges with regard to lacking interoperability. The existence of different IT-systems typically complicates data exchange [10] and can act as a barrier to inter-organisational collaboration.

*Lack of trust:* A lack of inter-personal trust typically impedes collaboration [64]. For instance, an atmosphere of distrust can lead to territorial behaviour and suspicions, hindering cooperation between healthcare professionals across organisations [12]. If one partner counteracts the common work repeatedly, there is a growing risk that distrust will develop and undermine an inter-organisational collaboration [64]. Furthermore, a lack of trust prompts partners to control the results (e.g. patient data) delivered by the collaborating organisation by collecting them again [59]. That costs both time as well as trust.

*Lack of mutual understanding:* Inter-organisational collaborations do not materialize, are hampered, or fail if one partner has little understanding of the goals, procedures and behaviour of the other(s). For instance, Loisel et al. [65] describe how, in the case of occupational rehabilitation, obstacles arose when different collaborating stakeholders such as the employer, the physicians or insurers had no understanding of the actions of the rehabilitation team, which delayed communication and hampered information exchange.

*Resistance to change:* Often, organisational members are not willing to accept changes connected to the implementation of inter-organisational collaboration, especially if they do not see the usefulness of the collaboration or fear the loss of their own professional existence [54]. Then managers of inter-organisational collaboration can face an unwillingness to change processes, to share knowledge, and to add to the collaboration. Ling and colleagues [10] report an unwillingness of general practitioners to engage in inter-organisational collaboration, as they had the feeling that change was forced upon them.

*Different professionalisation:* Within inter-organisational collaborations – more often than not – individuals with different professional backgrounds (e.g. physicians, nurses, managers educated in business schools) have to work together. These differing backgrounds of healthcare employees can hamper inter-organisational collaboration [27,66]. Established hierarchies between professions like physicians and nurses as well as power structures can be a constraint to inter-organisational collaboration. Furthermore, different professions underlie divergent cultural assumptions, professional values and follow different procedures. That can cause conflicts within inter-organisational collaborations involving inter-professional work [55,56]. Such conflicts can lead to the reaction that people start to defend their professional territory [48,51,67]. Furthermore, the incentive to fulfil tasks outside one’s own territory can be very low [55].

*Lack of communication:* Insufficient dialogue between organisational partners can impede collaboration, as the required knowledge transfer and exchange of information, for example, are hampered. What is more, a lack of communication can lead to ambiguities in responsibilities and regarding the aims of the collaboration [12,47].

### Barriers related to clinical practices

*Confidentiality issues:* Previous studies have reported that confidentiality issues can impede inter-organisational collaboration, e.g. leading to a lack of shared information about particular patients [26]. This barrier was especially mentioned in studies on integrated mental healthcare provision [25,26].

*Lack of information exchange:* Closely related and sometimes caused by confidentiality concerns, a lack of information exchange can hinder joint working across organisations. Such lacking information can, for instance, concern data on old test results and the medical regimen of clients during referrals [68]. This barrier can occur if some professionals involved in the collaboration have no access to certain data repositories [69]. A lack of information exchange can, in turn, increase the risk of errors and mistakes and, in certain areas such as that of mental health care, jeopardise the job security of employees [69].

## Discussion

The barriers identified in this literature review can be grouped into six main categories that stretch across different domains (see Figure 2). Because these categories serve mainly an analytical purpose, it is likely that the kinds of barriers and their domain of occurrence overlap (for instance, bad collaboration experiences can affect individuals entrusted with service delivery as well as organisations).

### Barriers and intentions

While reviewing the literature, it became clear that barriers can, on the one hand, be raised and actively and purposefully promoted by certain individual and organisational actors. On the other hand, barriers can also emerge more passively behind the back of actors, e.g. due to certain institutionalized structures [cf. also 37]. *Actively raised barriers* are deliberately activated by actors who want to sustain a desired state or prevent another and, hence, are similar to personal resistance to organisational change (e.g. an organisation that fears a loss of autonomy caused by collaboration). Johnson and colleagues [56] actually observed that organisational actors used complicated planning processes to delay joint working. Such barriers are more agent-driven and institutions recede into the background. *Passively emerging barriers* like historical developments or the existence of national borders, in contrast, are caused mostly by structural and institutional arrangements. They often have “historical roots” and can even be the result of path dependencies [70]. Actors who are willing to block collaboration deliberately can also strengthen these barriers on purpose. A further example of institutional arrangements that cause barriers to emerge passively is the professionalisation of different occupational groups with strong identities, which do not necessarily support the inter-professional collaboration that is often asked for when practicing inter-organisational collaboration in healthcare [48].

### Relationships between barriers and the influence of context

Closely related to the notion of barriers are questions relating to their causes [41] and interrelatedness, whereby the existence of one barrier may cause, influence, and/or reinforce other barriers. The reviewed literature provides insights on the relationships between barriers as well as their causes. However, for the following reasons, it is difficult to make generalizations about these relationships from the present sample of studies without developing a somewhat deterministic, contingent, and thereby non-realistic view [cf. e.g. 71]: although the relationships between barriers were analysed systematically, no consistent patterns were identifiable. In fact, almost each type of barrier seemed to be able to cause or influence other types of barriers, often in a recursive relationship to one another (causing chicken-egg problems). Three important ontological assumptions on society can help to explain this finding: first, it can be assumed that both agency (cf. more actively raised barriers) and structure (more passively induced barriers) are recursively related to each other, mutually (re-) producing and transforming each other [37]. Therefore, more structurally-rooted barriers are able to cause more agentic-driven barriers and vice versa. Second, and building on the first assumption, structures and actions – and thereby barriers – on different levels of analysis (ranging from macro to micro) influence each other. For instance, regulations (macro-level) may be a cause of conflict on matters of resources and interests between organisations (meso-level), which in turn may cause resistance to collaboration in every day practice (micro-level).

Third, every social action is context-dependent [37], whereby “context” includes not only national contexts, but also, for instance, formal and informal institutional arrangements, the voluntary or mandated nature of an inter-organisational collaboration [62], as well as former experiences and characteristic traits of individuals involved in an inter-organisational collaboration. However, the current studies included in the review (giving, for instance, little evidence on barriers in Asia, Africa, and South America as also relevant national contexts or characteristic traits of individuals) and the compilation of the types of barriers does not allow reliable conclusions on certain contingent patterns of contextual influence. For instance, the particular design of a national health system (e.g. public vs. private) does not exclude or favour the occurrence of certain types of barriers, as both kinds of health systems may require mandatory inter-organisational collaboration, which could cause resistance to this change.

**Figure 3:**
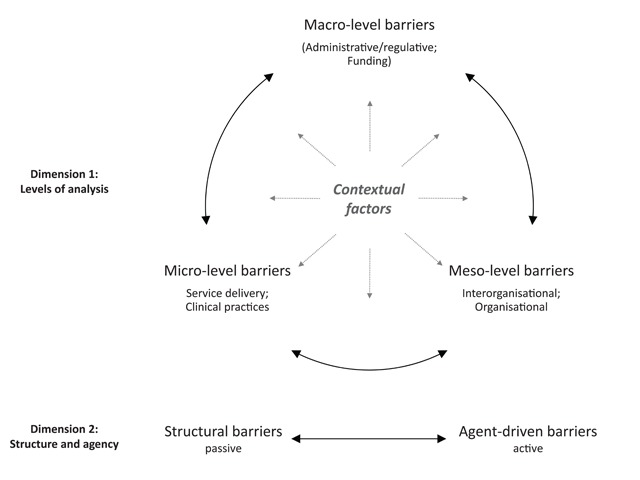
Relationships between barriers.

Following these assumptions, each type of barrier can cause and/or influence all other types of barriers, as barriers also influence each other on and across levels of analysis (dimension 1 in Figure 3) and independently of their roots in structure or agency (dimension 2 in Figure 3, see also section 6.1).

One example from the analysed studies highlights these multiple relationships between barriers: Johnson and colleagues [56] have highlighted barriers to the interorganisational collaboration between health and social care providers in Great Britain. They report that a main concern of the stakeholders involved in collaboration were costs – whose budget would pay for what. This represents the barrier “lack of organisational resources and funding” (*meso-level, agent-driven barrier*). Budget restrictions are often caused by regulations on the national regulative level, as also in this case (*macro level, structural barrier*). Additionally, hospital providers often have a good reason not to foster collaboration with community social care providers, as a following shift from patients to these providers would make hospital beds redundant, leading to a loss of resources for the hospitals (*organisational vs. collective interests, meso-level, agent-driven barrier*). Another relationship between barriers in this study concerns incompatible organisational structures (*meso-level, structural barrier*) and a lack of leadership and coordination (*meso-level, agent-driven barrier*): social services agencies were marked by less hierarchical management structures than health authorities, which often made the coordination of interorganisational collaboration difficult, for instance when setting goals and making decisions.

To sum up, when analysing barriers (either for research purposes or in order to overcome them), it seems helpful to assume that a barrier which is visible could be caused by one or several other barriers that are not obvious at first glance [41]. Taking a visible barrier as a starting point, it is advisable to look for related barriers which may prove to be the cause of the first barrier or influence it. Related barriers of this kind can also be found on other levels of analysis, and can be driven both by agents and/or structure.

## Conclusions

This paper contributes to previous research on barriers to integrated care in inter-organisational settings in various ways: first, this systematic review identifies twenty kinds of barriers that impede inter-organisational collaboration in six domains which are important to the integration of care. Thereby, barriers which can only be observed when different autonomous organisations intentionally aim to collaborate for the provision of integrated care are *systematically* highlighted. These barriers occur on the inter-organisational domain of analysis and differ from barriers reported in other settings such as markets or hierarchies. Second, the review illustrates that these barriers can either be actively raised by certain actors, or emerge more passively due to structural and institutional arrangements. Third, the context-dependence of barriers and their interrelatedness are discussed.

Based on this review, several areas for *further research* can be identified: first, our knowledge regarding barriers to the integration of care in inter-organisational settings would benefit from more systematic attention to existing organisation and network theories that address such barriers, even if only implicitly. While reviewing existing literature, it became clear that the notion of “barriers” lacks theoretical underpinning. For instance, the relationship between factors working as barriers to collaboration and the relationship to facilitators for collaboration (that help to lift the barrier) is still unclear. Second, empirical research should disentangle the interplay of barriers and their context-dependence more carefully, as well as their underlying causes and the visible symptoms [see also 41 for barriers to innovation]. For instance, it is very probable that the perceived mandatory or voluntary nature of an interorganisational collaboration will influence the actions of the organisations and individuals involved. The examination of such an approach to collaboration, however, requires diving deeply into specific cases. Further avenues for empirical research could also include exploring how network structure and governance [see e.g. 24] as parts of the context influence the existence of barriers and vice versa. Third, it seems vitally important to develop a comprehensive and more realistic understanding of the formation, development and/or failure of inter-organisational collaboration in the context of integrated care and the precise sources of barriers during this process. For instance, the same barrier may have a different effect at different stages of a collaboration (initiation, development, maturing), e.g. no effect on the collaboration, its transformation or even its termination. For instance, barriers in the formation phase of a collaboration can prevent collaboration before it even begins, one example being a lack of organisational resources and financial uncertainties [18,58]. Almost all of the works included in this literature review – as far as they are documented – deal with barriers during the implementation or later stages of a collaboration, and none of them gives reasons for the failure of a collaboration. Figure 2 can hence offer guidance on the analysis of barriers during the process of collaboration, but further empirical investigation is needed for its enhancement. Fourth, probably more barriers exist than those captured by the papers reviewed here. For instance, the risk aversion of decision makers, the “not invented here” phenomenon, or a lack of customer orientation [72] could act as further barriers. Fifth, empirical research should analyse how the existence of barriers to inter-organisational collaborations affects the outcome of integrated care, as barriers do not necessarily prevent or terminate collaboration, but merely slow down collaborative processes [20].

Like any review, this analysis has *limitations*. Because the literature search was focused mainly on peer-reviewed journals, some works meeting the inclusion criteria may have been left out (e.g. book chapters, monographs), thereby also omitting further barriers. Additionally, the review only includes the results of the reviewed studies, which could limit its scope (e.g. in terms of the range of existing barriers). And as the types of inter-organisational collaborations within the studies reviewed differ (e.g. with regard to the number and type of collaboration partners, the content and goal of collaborations and the national context), the comparability of the barriers arising in these inter-organisational collaborations underlies limitations. Hopefully, however, these findings will give new input to research and can help healthcare professionals, managers, teachers and policy makers to identify, avoid and overcome barriers to integrated care in inter-organisational settings.

## Additional File

The additional file for this article can be found as follows:

10.5334/ijic.3068.s1 Appendix Tables 1 and 2.
