# Supplementary material for: Barriers to the Integration of Care in Inter-Organisational Settings: A Literature Review
Source: Int J Integr Care. 2018 Jan 16;18(1):5. doi: 10.5334/ijic.3068 (PMC5887071; doi:10.5334/ijic.3068)
Supplement: Appendix — Tables 1 and 2. [file ijic-18-1-3068-s1.pdf]

APPENDIX

Table 1: Matched search terms

Search term…:	… represents different forms of inter-orga-nisational collaboration	… represents barriers	… represents integrated care settings	… excludes other forms of networks
Search terms	network*, consorti*, cluster*, alliance*, collabor*, cooperat*, partnership*	Barrier*, obstacl*, imped*, obstructi*, block*, inertia*, constrain*, problem*, inhibit*, rigid*	: "integrated care", "care coordinat*", "shared care", "coordinated care", "co-ordinated care", "comprehensive care", "seamless care", "transmural care".	inter-organisational, interorganisational, inter-organisational, inter-organisational, intersectoral
Application to:	Abstracts	Abstracts	Abstracts	Full texts

Table 2: Studies included in the systematic review

No.	Study	Journal	Design	Country	Type of collaboration	Barriers described after coding
1	Goldman 1982, [54]	American Journal of Psychiatry	Conceptual	-	Interorganisational cooperation between health and mental health services	Different professionalisation; historical developments; resistance to change
2	Hudson et al. 1997, [55]	Public Money & Management	Qualitative	England	Alliances between primary healthcare and social services	Cultural distance, different professionalisation; incompatible organisational structures; regulations
3	Bourdages et al, 2003, [60]	Health Promotion International	Qualitative	Canada	Collaboration of community stakeholders to set up integrated prevention programmes	Incompatible organisational structures; lack of leadership and coordination; missing actors
4	Van Hook and Ford 1998, [66]	Health & Social Work	Qualitative	U.S.	Collaboration between mental health staff and community health organisations	Different professionalisation; lack of leadership and coordination
5	Curtice et al. 2003, [73]	Journal of Interprofessional Care	Qualitative	Scotland	Collaboration between organizations providing health and social care	Different professionalisation, lack of leadership and coordination; incompatible organisational structures; lack of trust; power imbalances and conflicts; regulations
6	Glendinning 2003, [9]	Health Policy	Qualitative	England	Collaborations between general practitioners and community health services and between health and social services	Different professionalisation; lack of organisational resources and external funding; power imbalances and conflicts; regulations; resistance to change

7	Johnson et al. 2003, [56]	Journal of Interprofessional Care	Qualitative	Great Britain	Collaboration between health and social care providers	Different professionalisation; incompatible organisational structures; lack of leadership and coordination; lack of organisational resources and external funding; organisational vs. collective interests; regulations
8	Axelsson and Bihari-Axelsson 2005, [74]	Health Policy	Qualitative	Russian Federation	Intersectoral collaboration between different organizations	Organisational vs. collective interests
9	Loisel et al. 2005, [65]	Journal of Occupational Rehabilitation	Qualitative	Canada	Collaboration between an interdisciplinary and inter-organisational rehabilitation team	Lack of mutual understanding
10	Poland et al. 2005; [75]	Health & Social Care in the Community	Qualitative	Canada	Collaborations between communities and hospitals	Cultural distance; lack of organisational resources and external funding; regulations
11	Collins-Drogul 2006, [49]	Social Science and Medicine	Qualitative	U.S., Mexico	Collaboration between health organisations for providing cross-border healthcare	National borders
12	Dinesen et al. 2007, [61]	International Journal of Integrated Care	Qualitative	Denmark	Collaboration between a hospital and community health professionals to provide tele-homecare	Differences regarding collaboration design and aims; different professionalisation
13	Wihlman et al. 2008, [11]	International Journal of Integrated Care	Qualitative	Sweden	Inter-organisational vocational rehabilitation project	Different professionalisation; lack of communication; lack of leadership and coordination; organisational vs. collective interests
14	Axelsson and Axelsson 2009, [48]	Journal of Interprofessional Care	Qualitative	Sweden	Collaboration mainly between the health services, the social services and the social insurance administration in the field of vocational rehabilitation	Different professionalisation; organisational vs. collective interests
15	Kendall et al. 2009, [76]	Australian Journal of Primary Health	Qualitative	Australia	Regional collaborations of service providers to promote health	Former collaboration experiences; lack of organisational resources and external funding; organisational vs. collective interests
16	Wadman et al. 2009, [18]	International Journal of Integrated Care	Conceptual	Sweden, Denmark	Collaborations between primary and secondary healthcare	Cultural distance; historical developments; lack of organisational resources and external funding; organisational vs. collective interests
17	Hansson et al. 2010, [69]	International Journal of Integratedd Care	Qualitative	Sweden	Mental health coordination network	Lack of information exchange; lack of leadership and coordination
18	Pate et al. 2010, [51]	Journal of Health Organisation and Management	Mixed	Scotland	Partnerships within different networks having the character of mandated networks	Different professionalisation
19	Rivas et al. 2010, [77]	International Journal of Integrated Care	Qualitative	England	Collaborations between primary and secondary care	Organisational vs. collective interests
20	Stahl et al. 2010, [64]	Journal of Occupational Rehabilitation	Qualitative	Sweden	Inter-organisational collaboration between different agencies promoting return to work	Lack of trust; organisational vs. collaborative interests

21	Andersson et al. 2011, [12]	International Journal of Integrated Care	Literature Review	-	Collaboration between different agencies to enable vocational rehabilitation	Differences regarding collaboration design and aim; different professionalisation; lack of communication, lack of leadership and coordination; lack of trust; missing actors; organisational vs. collective interests; regulations
22	Dinesen et al. 2011, [27]	International Journal of Integrated Care	Qualitative	Denmark	Collaboration between hospital, and community health professionals and firms to provide tele-rehabilitation	Different professionalisation; incompatible organisational structures;
23	McDonald 2011, [78]	Journal of Interprofessional Carel	Qualitative	Australia	Collaborations across the public and private sector in diabetes care	Cultural distance; incompatible organisational structures
24	Stahl et sal. 2011, [21]	Journal of Occupational Rehabilitation	Qualitative	Sweden	Inter-organisational collaboration between different agencies promoting return to work	Regulations
25	Widmark et al. 2011, [79]	International Journal of Integrated Care	Qualitative	Sweden	Collaboration among professionals in health care, social services, and schools when handling health issue related to children	Different professionalisation; incompatible organisational structures; lack of communication; lack of leadership and coordination; lack of organisational resources and external funding; lack of trust
26	Ling et al. 2012, [10]	International Journal of Integrated Care	Qualitative	England	Pilot projects of integrated care including different forms of inter-organisational collaboration	Different professionalisation; lack of leadership and coordination; lack of technological standards; regulations; resistance to change
27	Stahl 2012, [80]	Journal of Occupational Rehabilitation	Qualitative	Sweden	Interprofessional teams working in interorganisational cooperation	Lack of trust, organisational vs. collective interests
28	Taylor-Robinson 2012, [81]	PloS one	Qualitative	England	Collaborations between different Primary Health Trust and Local Authorities	Cultural distance; lack of communication; lack of mutual understanding; regulations
29	Tsasis et al. 2012, [50]	International Journal of Integrated Care	Qualitative	Canada	Different models of integrated care; ranging from hierarchical to more collaborative models	Historical developments; lack of mutual understanding; lack of organisational resources and external funding; regulations
30	Zou et al. 2012, [62]	BMC Health Services Research	Qualitative	China	Collaboration between Centre for Disease Control and hospitals	Different professionalisation; organisational vs. collective interests
31	Kanste et. Al. 2013, [82]	International Journal of Integrated Care	Quantitative	Finland	Cooperation between health, welfare and education sectors serving children and families	Cultural distance; lack of leadership and coordination; lack of organisational resources and external funding
32	Anaf et al. 2014, [83]	Australian & New Zealand Journal of Public Health	Qualitative	New Zealand	Collaboration between primary health care organisations and organisations from other sectors (e.g. government, NGOs) to promote health outcomes	Lack of organisational resources and external funding; regulations
33	Carter et al. 2014, [84]	Implementation Science	Qualitative	England	Intra- and interorganisational collaboration for quality improvements in stroke care	Different professionalisation
34	Whiteford et al. 2014, [25]	Australian & New Zealand Journal of Psychiatry	Literature review	-	System-level intersectoral linkages involving mental health services and non-clinical support services	Confidentiality issues; lack of organisational resources and external funding; organisational vs. collective interests

35	Boothroyd et al. 2015, [47]	Journal of Behavioural Health Services & Research	Quantitative	U.S.	Collaboration between different professions, sectors and agencies in the field of children's mental health	Differences regarding collaboration design and aims; former collaboration experience; lack of communication; lack of organisational resources and external funding, regulations
36	Bang Christensen 2016, [59]	International Journal of Integrated Care	Qualitative	Denmark	Collaboration between municipalities, GPs and hospitals in the context of a telecare service	Different professionalisation; incompatible organisational structures; lack of trust; power imbalances and conflicts
37	Cooper et al. 2016, [26]	Child: Care, Health & Development	Literature review	-	Interagency collaboration across mental health services for children and young people	Confidentiality issues; cultural distance; different professionalisation; lack of communication; lack of leadership and coordination; lack of mutual understanding; lack of organisational resources and external funding; lack of trust
38	Lyngssø et al. 2016, [68]	International Journal of Integrated Care	Qualitative	Denmark	Collaboration of healthcare professionals caring for patients with chronic obstructive pulmonary disease	Cultural distance; lack of communication; lack of information exchange; lack of leadership and coordination; lack of technical standards
39	Behruzi et al. 2017, [67]	BMC Pregnancy & Childbirth	Qualitative	Canada	Interorganisational collaboration between midwives in birthing centres, and physicians and nurses in hospitals	Cultural distance; different professionalisation; incompatible organisational structures; lack of leadership; lack of organisational resources and external funding; organisational vs. collective interests; power imbalances and conflicts
40	McPherson et al. 2017, [58]	BMC Health Services Research	Qualitative	Canada	Cross-sectoral child health network	Power imbalances and conflicts

References

73. **Curtice, L, Stewart, A** and **Petch, A.** Moving towards integrated working in health and social care in Scotland: From maze to matrix. Journal of Interprofessional Care, 2003; 17(4): 335–50. DOI: https://doi.org/10.1080/1356182031000160817
74. **Axelsson, R** and **Bihari-Axelsson, S.** Intersectoral problems in the Russian organisation of public health. Health Policy, 2005; 73(3): 285–93. DOI: https://doi.org/10.1016/j.healthpol.2004.11.020
75. **Poland, B, Graham, H, Walsh, E, Williams, P, Fell, L, Lum, JM,** et al. 'Working at the margins' or'leading from behind'?: A Canadian study of hospital–community collaboration. Health and Social Care in the Community, 2005; 13(2): 125–35. DOI: https://doi.org/10.1111/j.1365-2524.2005.00542.x
76. **Kendall, E, Ehrlich, C, Young, M, Muenchberger, H, Wilkie, K** and **Rushton, C.** Health partnerships: Perspectives of medical practitioners in general practice, health systems and hospital settings. Australian Journal of Primary Health, 2009; 15(4): 319–25. DOI: https://doi.org/10.1071/PY09018
77. **Rivas, C, Abbott, S, Taylor, SJC, Clarke, A, Roberts, CM, Stone, R** and **Griffiths, C.** Collaborative working within UK NHS secondary care and across sectors for COPD and the impact of peer review: Qualitative findings from the UK National COPD Resources and Outcomes Project. International Journal of Integrated Care, 2010; 10(9): 1–13. DOI: https://doi.org/10.5334/ijic.573
78. **McDonald, J, Powell Davies, G, Jayasuriya, R** and **Fort Harris, M.** Collaboration across private and public sector primary health care services: Benefits, costs and policy implications. Journal of Interprofessional Care, 2011; 25(4): 258–64. DOI: https://doi.org/10.3109/13561820.2011.566650

79. **Widmark, C, Sandahl, C, Piuva, K** and **Bergman, D.** Barriers to collaboration between health care, social services and schools. International Journal of Integrated Care, 2011; 11(3): 1–9. DOI: https://doi.org/10.5334/ijic.653
80. **Stahl, C.** Implementing interorganizational cooperation in labour market reintegration: A case study. Journal of Occupational Rehabilitation, 2012; 22(2): 209–19. DOI: https://doi.org/10.1007/s10926-011-9337-x
81. **Taylor-Robinson, DC, Lloyd-Williams, F, Orton, L, Moonan, M, O'Flaherty, M** and **Capewell, S.** Barriers to partnership working in public health: A qualitative study. PloS one, 2012; 7(1): 1–8. DOI: https://doi.org/10.1371/journal.pone.0029536
82. **Kanste, O, Halme, N** and **Perälä, M-L.** Functionality of cooperation between health, welfare and education sectors serving children and families. International Journal of Integrated Care, 2013; 13(4): 1–15. DOI: https://doi.org/10.5334/ijic.1070
83. **Anaf, J, Baum, F, Freeman, T, Labonte, R, Javanparast, S, Jolley, G,** et al. Factors shaping intersectoral action in primary health care services. Australian & New Zealand Journal of Public Health, 2014; 38(6): 553–9. DOI: https://doi.org/10.1111/1753-6405.12284
84. **Carter, P, Ozieranski, P, McNicol, S, Power, M** and **Dixon-Woods, M.** How collaborative are quality improvement collaboratives: A qualitative study in stroke care. Implementation Science, 2014; 9(32): 1–11. DOI: https://doi.org/10.1186/1748-5908-9-32
